# Trends in Obesity, Overweight, and Attempted Weight Loss Among United States High School Students

**DOI:** 10.31486/toj.25.0111

**Published:** 2026

**Authors:** Jack Yang, Emily Krill, Cheila Llorens, Alan Kunz-Lomelin, Charles H. Hennekens, Panagiota Kitsantas

**Affiliations:** ^1^ Department of Population Health, Charles E. Schmidt College of Medicine, Florida Atlantic University, Boca Raton, FL; ^2^ Sandler School of Social Work, Florida Atlantic University, Boca Raton, FL; ^3^ Departments of Medicine and Population Health, Charles E. Schmidt College of Medicine, Florida Atlantic University, Boca Raton, FL; ^4^ Department of Health Administration and Policy, George Mason University, Fairfax, VA

**Keywords:** *Adolescent*, *body image*, *body mass index*, *diet–healthy*, *health risk behaviors*, *overweight*, *pediatric obesity*, *students*, *weight loss*

## Abstract

**Background:**

In the United States (US), obesity and overweight among adolescents present growing clinical and public health challenges. In this study, we examined trends in obesity, overweight, and attempted weight loss in a national sample of US adolescents.

**Methods:**

Data from the 2013 to 2023 Youth Risk Behavior Survey (YRBS) were used to explore these issues in a large sample of US high school students. Percentages were used as effect measures, and 95% confidence intervals were used to test statistical significance. Overall data are presented, as well as subgroup analyses by sex, race/ethnicity, and grade level.

**Results:**

Between 2013 and 2023, obesity rates increased from 13.7% to 15.9% (*P*<0.01), with the highest rates among Black and Hispanic/Latino adolescents and the lowest rate among Asian adolescents. In contrast, the percentage of overweight US high school students declined significantly from 16.6% to 14.7% (*P*<0.01), with a more notable decrease among male adolescents compared to female adolescents. The overall percentage of adolescents engaging in weight loss efforts declined from 47.7% in 2013 to 44.5% in 2023 (*P*<0.01). Declines were most notable among 10th and 12th graders. Female high school students reported higher rates of weight loss attempts than males.

**Conclusion:**

Data from the YRBS showed significant increases in obesity, declines in overweight, and fewer adolescents trying to lose weight. Although further research is necessary, these findings underscore the need for targeted clinical and public health strategies to reduce rising obesity rates and promote healthy behaviors among US high school students.

## INTRODUCTION

Approximately 1 in 5 or 22.2% of United States (US) adolescents aged 12 to 19 years are obese.^1^ According to the US Centers for Disease Control and Prevention (CDC), obesity in adolescents is defined as being in the ≥95th percentile relative to normative growth charts in body mass index (BMI) adjusted for age and sex. Overweight in adolescents is defined as BMI ≥85th percentile but <95th percentile.^2^ Being obese or overweight in adolescence poses considerable risks to both physical and psychological health, with consequences that extend into adulthood, including type 2 diabetes, hypertension, high cholesterol, obstructive sleep apnea, low self-esteem, and depression.^3^ Thus, the link between adolescent obesity and higher rates of morbidity and mortality in adults is not surprising.^4^ Obesity consequently poses major clinical and public health challenges during both youth and adulthood.

Understanding trends in adolescent obesity and overweight is critical for public health surveillance and for informing interventions. However, data on patterns of weight loss attempts remain limited, particularly when examined across subgroups defined by sex, race/ethnicity, and grade level. In this study, we examined trends in overweight and obesity, as well as attempted weight loss, among a national sample of US high school students.

## METHODS

Data for this study were obtained from the Youth Risk Behavior Survey (YRBS), a publicly available, cross-sectional, school-based survey conducted biennially by the CDC.^5^ The YRBS monitors health-related behaviors and experiences among adolescents that may lead to serious health risks, including injury or death. The survey targets students in grades 9 through 12 from both public and private high schools across all 50 US states and the District of Columbia.^6^ For our study, we focused on data collected from 2013 to 2023, with 2023 representing the most recent dataset available. The sample comprised US high school students from the following survey years: 2013 (n=13,324), 2015 (n=14,358), 2017 (n=13,146), 2019 (n=12,140), 2021 (n=14,896), and 2023 (n=17,814).

We used the following variables: (1) obesity, which was defined as students who were ≥95 percentile for BMI; (2) overweight, which was defined as adolescents with a BMI ≥85 percentile but <95 percentile; and (3) trying to lose weight, which was a response to a subjective question on the survey that students could answer with trying to lose weight, trying to gain weight, trying to stay the same weight, or not trying to do anything about their weight. The BMI percentile thresholds are based on the CDC BMI-for-age growth charts that have remained unchanged since their initial release in 2000 and are derived from national data collected between 1963 and 1994.^7^

We assessed trends in obesity, overweight, and self-reported attempts to lose weight overall, as well as among the subgroups of sex (male or female), race/ethnicity (White, Black, Asian, Hispanic/Latino), and grade level (9 to 12). Percentages were used as effect measures, and 95% confidence intervals were used to test statistical significance. The data used the appropriate YRBS sampling weights that account for school and student nonresponse as well as other features of the survey design.^6^

## RESULTS

**Figure 1.**
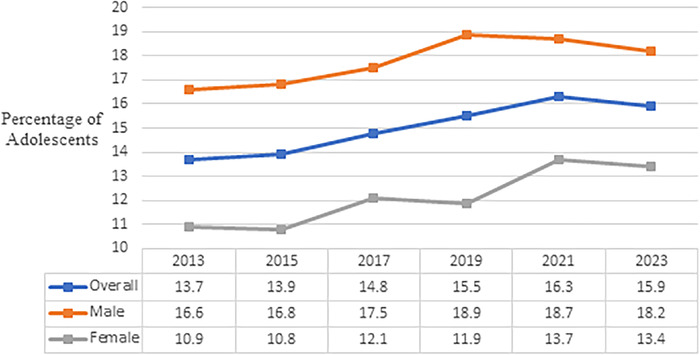
Obesity trends among United States high school students from 2013 to 2023, overall and by sex.

The overall percentage of adolescents classified as obese increased significantly from 13.7% in 2013 to 15.9% in 2023 (*P*<0.01) (Figure 1). This percentage rose steadily from 2013 to 2021, peaking at 16.3%, before experiencing a slight decline in 2023 to 15.9%. Among male adolescents, obesity rates increased consistently from 16.6% in 2013 to a peak of 18.9% in 2019 and then declined slightly to 18.2% in 2023. In contrast, female adolescents showed more fluctuation, with rates alternating between increases and decreases over the years. The lowest percentage among females was 10.8% in 2015, while the highest was 13.7% in 2021.

**Figure 2.**
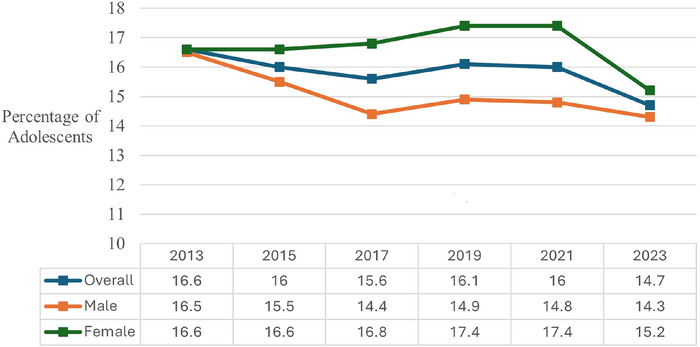
Overweight trends among United States high school students from 2013 to 2023, overall and by sex.

The overall percentage of adolescents who were overweight decreased significantly from 16.6% in 2013 to 14.7% in 2023 (*P*<0.01) (Figure 2). Both the overall percentage and the percentage for the male population decreased continuously from 2013 to 2017 before increasing in 2019, decreasing slightly in 2021, and dropping more significantly in 2023 to 14.7% overall and 14.3% for male adolescents. The female population stayed relatively consistent from 2013 to 2017 (around 16.6%), before experiencing a slight increase in 2019 and eventually decreasing in 2023 to 15.2%.

**Figure 3.**
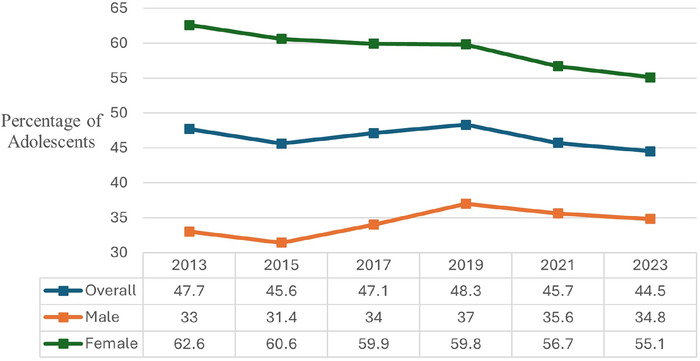
Self-reported attempts to lose weight trends among United States high school students from 2013 to 2023, overall and by sex.

The overall percentage of adolescents who were trying to lose weight decreased significantly from 47.7% in 2013 to 44.5% in 2023 (*P*<0.01) (Figure 3). The overall trend had an initial dip in 2015 (from 47.7% in 2013 to 45.6%) before rising to a peak in 2019 (48.3%) and then declining slightly in the subsequent years (to 44.5% in 2023). The percentage of females who were trying to lose weight significantly declined through the years, from 62.6% in 2013 to 55.1% in 2023 (*P*<0.01). Male adolescents were less likely than females to report trying to lose weight, with the highest percentage observed at 37% in 2019, followed by a decrease to 34.8% in 2023.

Black and Hispanic/Latino adolescents consistently had higher obesity rates compared to White and Asian adolescents, with Black adolescents reaching 21.2% in 2021 and Hispanic/Latino adolescents reaching 20.2% the same year (Table 1). Meanwhile, Asian adolescents had the lowest obesity prevalence, although the percentage rose notably from 5.6% in 2013 to 11.0% in 2023 (*P*<0.01). As noted previously, the overall percentage of youth trying to lose weight declined slightly over time, from 47.7% in 2013 to 44.5% in 2023, with Hispanic/Latino adolescents consistently showing the highest rates of weight loss attempts, peaking at 55.4% in 2017, and White youth showing a significant gradual decrease in such efforts, from 47.1% in 2013 to 41.0% in 2023 (*P*<0.01).

**Table 1.** Obesity, Overweight, and Trying to Lose Weight in United States Adolescents by Race/Ethnicity

Variable / Subgroup	2013, n=13,324	2015, n=14,358	2017, n=13,146	2019, n=12,140	2021, n=14,896	2023, n=17,814
**Obesity**						
White	13.1 (11.6-14.9)	12.4 (10.5-14.6)	12.5 (10.9-14.3)	13.1 (11.3-15.2)	13.7 (12.0-15.6)	13.4 (12.4-14.4)
Black	15.7 (13.8-17.8)	16.8 (14.2-19.6)	18.2 (16.3-20.3)	21.1 (17.7-24.8)	21.2 (18.5-24.3)	20.2 (16.9-24.1)
Asian	5.6 (3.5-9.0)	5.5 (3.6-8.4)	7.9 (5.1-12.0)	6.5 (3.8-10.7)	7.7 (5.0-11.7)	11.0 (7.5-15.9)
Hispanic/ Latino	15.2 (13.1-17.5)	16.4 (14.8-18.2)	18.2 (16.9-19.5)	19.2 (16.7-21.9)	20.2 (17.9-22.8)	19.5 (17.9-22.4)
**Overweight**						
White	15.6 (14.1-17.3)	15.2 (14.1-16.5)	14.0 (12.8-15.3)	14.6 (13.1-16.2)	14.6 (13.5-15.7)	13.5 (12.3-14.7)
Black	19.1 (17.2-21.3)	17.2 (15.1-19.5)	17.8 (15.4-20.5)	16.4 (13.5-19.6)	18.6 (17.0-20.5)	17.5 (14.9-20.4)
Asian	13.6 (10.1-18.1)	11.9 (9.1-15.5)	13.4 (10.4-17.3)	11.0 (8.8-13.6)	8.3 (4.9-13.6)	11.1 (8.6-14.4)
Hispanic/ Latino	18.3 (16.8-19.8)	18.4 (17.0-19.9)	19.5 (18.0-21.0)	19.6 (17.4-22.0)	20.9 (19.7-22.1)	16.2 (14.5-18.1)
**Trying to lose weight**						
White	47.1 (44.9-49.3)	44.1 (41.8-46.4)	45.1 (42.9-47.2)	46.6 (43.7-49.6)	42.7 (40.7-44.8)	41.0 (38.6-43.5)
Black	40.9 (38.5-43.3)	39.4 (35.7-43.2)	42.3 (39.4-45.3)	40.1 (34.6-45.9)	45.7 (42.8-48.7)	42.3 (37.9-47.3)
Asian	47.8 (43.8-51.8)	45.3 (40.2-50.5)	48.7 (44.0-53.5)	47.5 (39.3-55.8)	42.2 (30.4-56.3)	41.4 (35.7-47.3)
Hispanic/ Latino	54.5 (51.9-57.0)	53.1 (50.2-55.9)	55.4 (53.4-57.4)	54.9 (52.8-56.9)	53.6 (50.5-56.8)	52.4 (50.0-54.7)

Note: Data are reported as % (95% confidence interval).

Table 2 shows that 9th and 12th graders had the lowest overweight prevalence in 2023 at 14.4% and 12.9%, respectively, representing a statistically significant decrease from 2013 (*P*<0.05). In 2023, 11th graders had the highest obesity prevalence at 17.3%, representing a significant increase from 14.5% in 2013 (*P*<0.05). Similarly, 9th graders also showed a significant increase in obesity compared to 2013 (*P*<0.05). The most significant declines among adolescents trying to lose weight occurred among 10th and 12th graders (*P*<0.05). Overall, while obesity increased slightly over the decade, efforts to lose weight decreased, particularly among older adolescents.

**Table 2.** Obesity, Overweight, and Trying to Lose Weight in United States Adolescents by Grade Level

Variable / Subgroup	2013, n=13,324	2015, n=14,358	2017, n=13,146	2019, n=12,140	2021, n=14,896	2023, n=17,814
**Obesity**						
9th grade	13.2 (11.8-14.9)	13.0 (11.7-14.4)	13.1 (11.5-14.9)	14.8 (12.8-17.1)	16.1 (14.4-17.9)	16.3 (14.1-18.7)
10th grade	13.6 (11.8-15.7)	15.2 (13.0-17.6)	14.9 (13.0-17.0)	15.9 (13.3-18.8)	16.6 (14.8-18.5)	14.8 (13.2-16.5)
11th grade	14.5 (12.9-16.4)	14.5 (12.6-16.6)	16.9 (15.3-18.7)	15.7 (13.6-18.1)	15.3 (13.6-17.2)	17.3 (15.4-19.5)
12th grade	13.5 (11.7-15.5)	12.7 (10.7-15.1)	14.2 (12.4-16.2)	15.5 (13.6-17.7)	17.3 (15.5-19.3)	15.3 (13.7-17.0)
**Overweight**						
9th grade	18.2 (16.2-20.5)	16.8 (14.8-19.0)	15.7 (14.3-17.2)	15.7 (13.7-17.8)	17.4 (15.8-19.2)	14.4 (12.8-16.2)
10th grade	16.1 (14.7-17.6)	15.5 (13.8-17.4)	16.2 (14.3-18.3)	16.6 (14.9-18.5)	15.6 (13.9-17.4)	15.8 (14.4-17.2)
11th grade	15.6 (14.1-17.3)	15.9 (14.5-17.4)	16.5 (14.8-18.2)	16.6 (14.4-19.2)	16.8 (15.3-18.4)	15.5 (14.1-17.1)
12th grade	16.2 (14.4-18.1)	16.0 (13.8-18.4)	14.0 (12.0-16.3)	15.5 (13.6-17.7)	14.2 (12.7-15.7)	12.9 (10.9-15.2)
**Trying to lose weight**						
9th grade	48.7 (45.9-51.6)	44.3 (41.7-47.0)	46.2 (44.1-48.4)	47.2 (44.3-50.2)	47.4 (44.4-50.4)	44.9 (41.7-48.1)
10th grade	46.7 (43.6-50.0)	45.7 (43.1-48.4)	46.3 (44.4-48.3)	50.7 (47.7-53.7)	43.1 (39.7-46.6)	42.7 (40.2-45.3)
11th grade	48.6 (46.3-50.9)	45.7 (42.3-49.2)	48.6 (45.9-51.3)	48.2 (45.8-50.6)	46.1 (42.8-49.5)	46.4 (43.3-49.5)
12th grade	47.0 (44.9-49.1)	47.3 (44.6-50.0)	47.8 (45.2-50.5)	47.1 (43.7-50.4)	46.4 (44.3-48.6)	43.9 (41.0-46.9)

Note: Data are reported as % (95% confidence interval).

## DISCUSSION

In this study, obesity among US adolescents increased significantly, while overweight declined (*P*<0.01). Between 2013 and 2023, obesity increased from 13.7% to 15.9%, with the highest rates observed among Black and Hispanic/Latino adolescents and the lowest rate among Asian adolescents. In contrast, the percentage of overweight US high school students declined significantly from 16.6% to 14.7%, with a more notable decrease among male adolescents vs female adolescents. The overall percentage of weight loss efforts among adolescents also declined from 47.7% in 2013 to 44.5% in 2023. Declines were most notable among 10th and 12th graders. Female high school students reported higher rates of weight loss attempts than males. Taken together, the data portray a population in which heavier body weights are becoming more common, while motivation, especially among females, to engage in weight control is waning.

Overall, these findings are compatible with other recently published data (2024) that indicate a rising percentage of obesity in US adolescents.^8^ Trends in attempted weight loss by gender warrant further examination. The findings of this study show that female adolescents report attempts to lose weight at much higher rates than males but are also less likely to report trying to lose weight compared to earlier years. This pattern is consistent with recently published data (2024) indicating that body dissatisfaction and dieting behaviors among adolescent females have been decreasing during the past decade,^9^ which may, in turn, contribute to fewer reported weight-loss attempts.

The slight male increase in trying to lose weight between the years 2015 and 2019 also warrants further research. This increase is plausibly related to males seeking more muscular or athletic body builds.^10^ Further analytic studies are necessary to elucidate how perceived gender roles and weight-related stereotypes influence attitudes toward attempting to lose weight and the perceived importance of losing weight among youth over time. Policymakers and intervention developers may wish to consider incorporating gender-based approaches for educating adolescents on specific perceptions and behaviors.^11^ In addition, strategies that reinforce body satisfaction and address shifting social norms, particularly declines in body dissatisfaction and dieting behaviors among adolescent females, may increase the likelihood of engaging adolescents in healthy, long-term weight management.

Overall, all racial/ethnic groups had marked increases in obesity, and the percentages of obesity were consistently highest among Hispanic/Latino and Black adolescents, with both groups demonstrating increases in prevalence from 15.2% of Hispanic/Latino and 15.7% of Black adolescents in 2013 to 19.5% and 20.2% in 2023, respectively. In contrast, the overweight percentages decreased across all racial/ethnic groups, but Black and Hispanic/Latino adolescents still had higher percentages of overweight compared to White and Asian adolescents in 2023. These observations are consistent with previous research that suggests Hispanic/Latino and Black adolescents are at increased risk for obesity because of socioeconomic and environmental factors such as lack of access to healthy food options, varying health literacy levels, and lower levels of physical activity.^12^ Public health policies should aim to improve access to healthy foods by reducing the proximity of fast food restaurants near schools in low socioeconomic areas and promoting grocery store development.^13^ Furthermore, increasing accessibility to effective public transportation and educating the public on healthy exercise habits could increase weight loss success among all racial groups.^14^ Future research could explore how culturally tailored interventions, attention to structural barriers, and preventive health care can play a role in addressing disparities and optimizing health outcomes for adolescents across all racial and ethnic groups.

The findings from this study also highlight the need for comprehensive school-based strategies that target nutrition literacy, body image perceptions, and mental health among adolescents of high school age, independent of grade level. Putting these strategies into action could tackle misconceptions about healthy weight that have been ingrained since an early age and promote realistic body expectations.^15^

Given that obesity arises from multiple contributing factors, implementation of a range of public health policies that promote weight loss motivation among obese and overweight adolescents is important. With male adolescents in the YRBS data showing the highest obesity rates and female adolescents showing decreased engagement in weight loss efforts, federal and state initiatives such as the CDC State Physical Activity and Nutrition program could consider disaggregated targets and reporting while providing content tailored to the specific needs and perspectives of each group.^16^ In addition, such programs could integrate culturally relevant dietary guidance and community-based activity opportunities that may help motivate engagement among adolescents.

In clinical settings, extending the Medicaid Early and Periodic Screening, Diagnostic and Treatment benefits to cover multidisciplinary, teen-specific weight management groups, including mental health and media literacy components, could create a continuum of care for youth whose needs exceed prevention programs.^17^ Recent research (2020, 2021) also advocates for nurse practitioners and other health care providers to discuss healthy habits with families, such as lower sugar intake and recommended activity levels, that could lead to the early establishment of healthy habits in adolescents and eventually have a lasting impact on their adult weight status.^18,19^

Schools and communities can reinforce these policy actions. One potential strategy is the introduction of comprehensive nutrition education courses in high schools. Many schools lack programs that effectively inform students about the risks associated with unhealthy lifestyles and the benefits of proper dietary habits. Providing students with this foundational knowledge could empower healthier decision-making.^20^ Another possibility is to offer students subsidized or complimentary memberships to local fitness facilities. Access to such resources may encourage participation in physical activity, particularly aerobic exercise, that can significantly reduce BMI and improve fitness levels among adolescents.^21^

A major strength of this study is that the data are a representative US sample. A limitation of the study is that we did not have access to individual-level data and so were unable to conduct the preferred statistical analysis to look deeper into the shift in obesity or to stratify. Another limitation is the reliance on self-reported measures collected through the YRBS. Self-reported data are susceptible to reporting biases, including the underestimation of weight or overestimation of height, that may affect the accuracy of the findings. An additional limitation is the timing of the data collection. The YRBS is administered every 2 years during the spring; in 2021, however, data collection occurred during the fall because of disruptions caused by the coronavirus disease 2019 pandemic. This shift in timing and student experiences during the pandemic may or may not have introduced variability. Finally, the cross-sectional design of this study does not allow for assessment of longitudinal progression from overweight to obesity and other measures within a single cohort. As with all observational, cross-sectional analyses, these findings reflect statistical associations rather than causal relationships and should be interpreted in light of potential unmeasured confounding and other limitations.^22,23^ Further analytic studies should address whether students attempting to lose weight are engaging in healthy practices, such as increased physical activity and balanced dieting, or resorting to unhealthy methods such as restrictive eating or diet pill usage.

## CONCLUSION

This study highlights a shift in US adolescent weight trends from 2013 to 2023. Although overweight prevalence declined, this decline was largely offset by rising obesity rates. Notably, fewer female adolescents reported attempting to lose weight, potentially reflecting broader declines in body dissatisfaction and dieting behaviors. These findings underscore the need for targeted, culturally sensitive, and gender-specific public health interventions. Schools, health care providers, and community organizations should collaborate to promote healthy weight management that emphasizes positive body image, balanced nutrition, and regular physical activity. Ongoing surveillance and research are essential to monitor trends and to ensure that interventions remain effective across diverse adolescent populations.

## Notes

*This article meets the Accreditation Council for Graduate Medical Education and the American Board of Medical Specialties Maintenance of Certification competencies for Patient Care, Medical Knowledge, and Systems-Based Practice.*
